# Does contemporary Western culture play a role in mental disorders?

**DOI:** 10.3389/fpsyt.2022.978860

**Published:** 2022-09-07

**Authors:** Dimitri Marques Abramov, Paulo-de-Tarso de Castro Peixoto

**Affiliations:** ^1^Laboratory of Neurobiology and Clinical Neurophysiology, National Institute of Women, Children and Adolescents' Health Fernandes Figueira, Oswaldo Cruz Foundation, Rio de Janeiro, Brazil; ^2^Open College and Laboratory of Emotions, Affections, Society & Subjectivities, Office of Higher Education, Macaé City Hall, Macaé, Brazil

**Keywords:** Western culture, Ubuntu, individualism – collectivism, materialism, epidemiology of mental disorders, neurodiversity

## Introduction

Mental illnesses are entities with a multidimensional nature: aside from constitutional/endogenetic determinants, social and cultural dimensions also seem to be determinants of mental health (1). In the present study, we will focus on the sociocultural aspect of Mental Health, which might explain the existence of a heterogeneous prevalence of mental disorders among different countries, as shown by epidemiological evidence.

Cross-national surveys have broadly adopted similar diagnostic tools, based on either DSM or ICD. There is fair criticism concerning the indiscriminate applicability of these nosological systems in different cultures, as DSM-5 itself warns us (2). Moreover, the adoption of different methodologies in these surveys produces biases that also limit the validity of comparisons; the DSM or ICD criteria somehow map the symptoms of mental distress to a certain extent, regardless of the validity of the diagnostic categories and the universality of their application.

Here, we raise some aspects of contemporary Western culture as a putative correlate of mental distress by comparatively discussing cultural characteristics of countries that show discrepancies in the prevalence of mental disorders. Our interest lies in the large gray area of subjective suffering, regarded as depressive and anxiety disorders, where epidemiological data show the highest cross-cultural divergence.

## From epidemiological evidence

Perhaps the most important epidemiological study on global prevalence of mental disorders is that by Kessler et al. (3), from the World Mental Health Survey Initiative. This study was expected to enable a comparative assessment of the prevalence of mental disorders among different countries with minimal bias.

If mental disorders, as defined by DSM, were equally significant across different cultures, and if they were primarily endogenous and genetically determined processes, similar prevalences of mental disorders could be expected among different countries in large samples. However, that is not what the study shows. While 47.4 and 39.3% of the populations from the USA and New Zealand, respectively, have a history of mental disorder, Nigeria shows the lowest prevalence (12.0%) among countries. Other epidemiological studies somehow confirm the low prevalence of mental disorders, e.g., depression and anxiety, in African countries compared to the Western world (4–6).

How do we explain that Nigeria, which has one of the worst HDI's in the world, had the best mental health status in the study samples? African communities, especially Nigeria, consider mental suffering a taboo and they are opposed to understanding this experience as a health disorder, which can negatively affect the verification of these statistics (7, 8). Kessler et al. (3) are also cautious in their explanations, assuming that there could have been potential errors in countries “with no strong tradition of independent public opinion survey”, where under-reporting might have occurred. However, these limitations do not apply to countries such as Japan or Israel, which have a long tradition of individual freedoms and consolidated scientific culture, which have also shown lower prevalence values than western countries (3).

Supporting the validity of these studies, a similar prevalence of bipolar disorder, a condition with important genetic determination, has been reported between Africa and Western countries (4). Indeed, the understanding of subjective experiences as well as the particularities of social organization across different cultures can be confounding factors in the diagnosis of depressive and anxiety disorders, which would explain the discrepancy in prevalence found in this study (3–6). This can be observed in social anxiety disorder (9). In fact, the influence of the subjective experience or social organization on the construction of diagnoses is natural evidence in itself of the determining role played by culture in mental suffering.

The WMH survey (3) specifically explored substance use disorders and showed that the lifetime prevalence in Nigeria was 3.7%, while it was 14.6% in the USA (3). Despite the complex determinants of substance use disorders, their diagnosis is mostly based on behavioral phenomena rather than subjective experiences (10). The diagnosis should thus be more objective and less sensitive to bias caused by the cultural understanding of subjective experiences. Thus, discrepancies in the prevalence of addictions corroborate the association between mental suffering and Western culture.

Another objective marker of mental suffering is suicide, a serious outcome of many mental disorders. Its incidences are higher in Western societies (11), which somewhat corroborates the findings of Kessler et al. (3): while USA reports 14.40 to 21.27 suicides per 100,000 people between 15 and 65 years old (crude rates), Israel and Nigeria have shown rates of 3.22–9.38 and 1.83–14.65, respectively, in the same age group (12). Under-notification should not be a plausible explanation for Nigerian data, since their crude suicide rates among the elderly are much higher than in the USA (12).

Therefore, the DSM classification could be universally valid for cross-cultural evaluation, since the understanding of subjective experiences, important in many diagnostic criteria, only confirms the prominent role played by culture in comprising many types of mental suffering. We need to know which cultural differences could be correlated with each suffering.

## Ubuntu

A universal paradigm shared by cultures throughout the African continent is the “Ubuntu,” which is not present in Western cultures, and could explain the unexpected epidemiological findings mentioned above. For all peoples in Africa, despite their dialectic peculiarities, the idea of a “self” does not lie within the scope of individuality (13–15). Unfortunately, there are very few empirical studies on Ubuntu.

Kpanake (13) reported that Ubuntu, which is one fundamental value system promoted by many African cultures, “refers to African values of collective relatedness, interdependence, communality, group solidarity, and conformity” (...) “the self is perceived in relation to the group; that is, individuals are perceived not as entities that are independent from one another, but as part of an interdependent communal system”; the problems and demands of one person are problems and demands of everyone. According to Ubuntu, persons depend on other persons to be persons; this means that life only makes sense through relationships, not only between living people, but also with the gods, the ancestors, and Nature itself (13–15). Thus, the human being is never alone.

The Bantu society has a life-force epistemology, which is a thread of life that connects, binds, and intertwines beings, thus comprising one single body (16). The expression “Ubuntu” follows this perspective. The life force of each being is the thread of life that binds each individual in the formation of the peoples, societies, and nature. Thus, Ubuntu is the experience of one joining others in a community.

Despite cultural westernization, it is possible that Ubuntu still survives at the core of African people. Akiwowo (17), informed us that many Nigerian tribes have assimilated foreign cultures, yet have not lost their own cultural identity, and when their individuals move to large urban centers, they take the tribe with them.

Perhaps the social and spiritual collectivism could be a paradigm of any tribal culture far beyond the African continent, which has emerged spontaneously from the biopsychosocial nature of the *Homo sapiens* in their evolution, and has survived in other civilizations. In Eastern societies, the prevalence of mental disorders was almost one third of those in USA (3). Eastern culture is regarded as essentially collectivistic (11, 18). Somehow, this collectivism might also be present in the Israeli kibbutz (19).

## The contemporary Western culture

Enlightenment has provided contemporary Western culture with the ideals of “individual” rights and freedom, scientific knowledge (which is reductionist), and the material universe (19). As opposed to the natural emergency of a self-organizing tribal culture over thousands of years, contemporary society results from the institution of philosophical, scientific, aesthetic, and political projects that determine our most fundamental values (20, 21). This Enlightenment project appears to have resulted in individuals who have collapsed into scientific objectivism and materialism, with strict boundaries around themselves and their properties, which would lead to a real health hazard (22). The Republican spirit has brought an important and indisputable evolution for civilization, which is the dawn of modern democracy and constitutional states. However, the sense of community in Western culture seems to be essentially different from Ubuntu: historically, “the identity of a collective in American culture is not associated to a united whole (Ubuntu), but to the idea of an ‘aligned' many” (23).

What has resulted from this Western project? Unfortunately, we see the devitalisation of the subjectivity encapsulated in individualism, directly or indirectly related to mental illness. People have lost their ancestors, their territories, their gods, and assume a private life where they find themselves helplessly entangled in their problems. This feeling of disconnection with the social world, of not belonging to yourself, of not belonging to coexistence bonds, of not belonging to a broader community, would engender the experience of a non-existence (24). Loneliness rises as a phenomenon of a society focused on performance, results, profit, competitiveness, and individualism. On the other hand, the primordial experience of Ubuntu seems to comprise a system of affective and psychosocial references with protective roles for mental health in face of life's obstacles (25).

Clinical evidence supports this viewpoint: loneliness is regarded as a public mental health issue (26). Remarkably, it also appears to be a risk factor for suicidal behavior, which indicates its role in mental distress (27–29). Regardless of its nature or classification, relief and resolution of mental suffering have been correlated with social support (30).

## Discussion

Mental health is a complex field, where several factors are inextricably integrated to determine mental wellbeing, and the reductionist biomedical paradigm fails to grasp the human complexity that should be fully considered in clinical psychiatric practice (31). Instead, clinical practice is focused on psychopharmacology, which probably mitigates the biological response to the stress produced by subjective suffering (32). Mental disorders can be related to the stress caused by our social relationships, cultural values, and collective beliefs, which could affect both typical and neurodiverse subjects (33–38).

Regarding endogenetic predispositions [which might only determine neurodiversity (39) rather than primary mental disorders], cultures manifest values specific to each population and might integrate common characteristics and behaviors dimensionally. This could lead to the high diversity of collective manifestations of mental suffering. Thus, according to the epidemiological, sociological, and clinical evidence shown here, the cultural dimension seems to play a determining role in mental health.

Therefore, the high incidence of many mental problems in Western countries might be centered on some contemporary values of their culture, which might be foreign to human nature and human neurodiversity. At the core of this problem would lie individualism and materialism, which might even admit a collective alignment, yet the experience of a collective existence has been lost. On the other hand, Italy and Spain have shown low prevalence of mental disorders compared to other Western countries, which suggests the existence of other cultural factors that might protect mental health (3). A recent systematic review indicates an increase in incidence of mental suffering among African adolescents (40), thus indicating a potentially changing setting.

We here hypothesize that collective experience still lives on through Ubuntu in Africa (and putative similar cultural complexes that are organic to other cultures), and that would be one of the reasons for the epidemiological evidence shown here. However, this hypothesis needs to be empirically checked through cross-cultural observation and by assessing methodologies that might bring back the collective experience of Ubuntu. One example would be Urban Heterogenesis (24, 41). It represents a practical epistemology that constitutes mental healthcare at public places around the city. It combines social diversity, building “healthcare communities” to replicate the spirit of tribes. The perspective of “vital force” in the Bantu philosophy is combined to Spinoza's life force, through his concept of “conatus” (the desire to persevere in existence). In Urban Heterogenesis, a “collective conatus,” which manifests as mental health, autopoietically emerges from the community of diverse people.

We believe it is paramount and urgent to invest in the understanding of mental suffering from these social and cultural perspectives. We should invest much more in the promotion of mental health that goes beyond psychopharmacology, which perhaps should have only a supportive role.

Humankind surely needs to discover its true nature and live according to it. Furthermore, this nature seems to be living in an organically indissociable collective, which is far from Western individualism and materialism. Perhaps we should place some of our deepest values into question.

## Author contributions

All authors listed have made a substantial, direct, and intellectual contribution to the work and approved it for publication.

## Conflict of interest

The authors declare that the research was conducted in the absence of any commercial or financial relationships that could be construed as a potential conflict of interest.

## Publisher's note

All claims expressed in this article are solely those of the authors and do not necessarily represent those of their affiliated organizations, or those of the publisher, the editors and the reviewers. Any product that may be evaluated in this article, or claim that may be made by its manufacturer, is not guaranteed or endorsed by the publisher.
